# Optimization of dental implant imaging using photon-counting detector computed tomography in oral and maxillofacial surgery: an *ex vivo* study

**DOI:** 10.1186/s41747-026-00710-5

**Published:** 2026-04-13

**Authors:** Adib Al-Haj Husain, Tristan T. Demmert, Konstantin Klambauer, Philipp Maintz, Thomas Flohr, Harald Essig, Maximilian Eberhard Hermann Wagner, Sameena Sandhu, Egon Burian, Bernd Stadlinger, Hatem Alkadhi, Victor Mergen

**Affiliations:** 1 Department of Cranio-Maxillofacial and Oral Surgery, University Hospital Zurich, University of Zurich, Zurich, Switzerland; 2 Department of Cranio-Maxillofacial Surgery, GROW School for Oncology and Reproduction, Maastricht University Medical Centre, Maastricht, The Netherlands; 3 Diagnostic and Interventional Radiology, University Hospital Zurich, University of Zurich, Zurich, Switzerland; 4 Department of Radiology and Nuclear Medicine, Maastricht University Medical Centre, Maastricht, The Netherlands; 5 Clinic of Cranio-Maxillofacial and Oral Surgery, Center of Dental Medicine, University of Zurich, Zurich, Switzerland

**Keywords:** Artifact reduction, Dental implants, Image artifacts, Photon-counting detector computed tomography, Virtual monoenergetic imaging

## Abstract

**Objective:**

To determine optimal reconstruction parameters for dental implant imaging using photon-counting detector CT (PCD-CT), including ultra-high-resolution (UHR) images, virtual monoenergetic images (VMI), and iterative metal artifact reduction (iMAR).

**Materials and methods:**

In this *ex vivo* study, six pig mandibles were prepared with two titanium-based implants and imaged on a PCD-CT. Scans were reconstructed as UHR images and VMI from 70–190 keV at 10 keV increments with and without iMAR. Two independent readers qualitatively evaluated image quality and artifact severity using five-point visual rating scales (5 = excellent, no or minimal artifacts; and 1 = very poor, non-diagnostic, severe artifacts). Two readers quantified artifact severity, defined as the standard deviations in attenuation in regions of interest adjacent to the implants.

**Results:**

UHR images without iMAR yielded high image quality (median 5 for both readers) with minor artifact severity (median 4 for both), whereas iMAR reduced image quality (median 3 for both). VMI without iMAR showed decreasing artifacts at higher energy levels. VMI at 120–130 keV achieved optimal image quality (median 5 for both readers at 120 keV, and medians 4 and 5 at 130 keV) with minimal artifacts (median 5 for both), whereas iMAR reduced quality. Quantitative artifact burden decreased with higher energy levels (from 200 HU at 70 keV to 113 HU at 190 keV), and no improvement was observed using iMAR.

**Conclusions:**

PCD-CT effectively reduces metal-induced artifacts in dental implant imaging, with UHR images and VMI at 120–130 keV providing optimal image quality, while reconstructions with iMAR offered no further benefit.

**Relevance statement:**

PCD-CT provides excellent dental implant visualization while minimizing the impact of metal artifacts.

**Key Points:**

- In this *ex vivo* study, ultra-high-resolution images and virtual monoenergetic images at 120–130 keV from PCD-CT effectively reduce metal artifacts from dental implants.
- Effective artifact reduction offers excellent visualization of the bone-implant interface.
- Iterative metal artifact reduction (iMAR) did not provide additional benefit for visualization of the bone-implant interface.

**Graphical Abstract:**

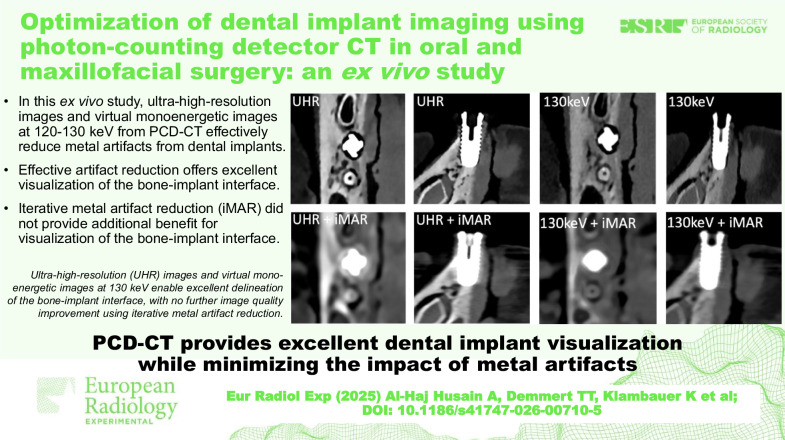

## Background

Dental implant imaging has become an essential component in routine clinical practice, supporting perioperative assessment, long-term follow-up, and the evaluation of oral health-related pathologies in the vicinity of the titanium-based implants [1].

Cone-beam computed tomography (CBCT) is widely recognized as the reference standard imaging modality in oral and maxillofacial surgery for evaluating implant-related diagnostic challenges [2]. This is largely attributed to its widespread accessibility, relatively low cost, and superior spatial resolution compared to conventional energy-integrating detector CT systems [3]. Despite these advantages, the widespread use of CBCT across various dentomaxillofacial applications and its decisive role in image-guided clinical decision-making present certain limitations, such as beam hardening and metal-induced artifacts, which can impair the accurate depiction of the surgical site and may not allow for the detection of complications and newly arising adjacent pathologies during long-term follow-up [2, 4].

Photon-counting detector computed tomography (PCD-CT) represents the most recent advancement of CT technology and has emerged as a highly promising modality to overcome several shortcomings of CBCT [5–9]. The commercially available PCD-CT enables data acquisition at 120 × 0.2 mm collimation and the reconstruction of both ultra-high-resolution (UHR) images with a slice thickness of 0.2 mm and virtual monoenergetic images (VMI) with a slice thickness of 0.4 mm at varying energy levels from the same scan data, with the additional option of applying advanced iterative metal artifact reduction (iMAR) algorithms [8, 10–12]. Initial studies have indicated the effectiveness of PCD-CT in reducing artifacts from dental implants or restorations in head and neck scans [6, 13, 14], as well as in postoperative maxillofacial trauma assessment [15, 16]. However, evidence supporting the use of PCD-CT for dedicated dental implant imaging remains limited, particularly in applications requiring precise evaluation of the implant-bone interface and detailed visualization of adjacent anatomic structures [17, 18]. Such precision is essential across a range of diagnostic and preoperative scenarios, for example, when assessing implant osseointegration, investigating suspected postoperative peri-implant complications, or planning anatomically challenging surgical procedures in which the proximity to the mandibular canal must be carefully evaluated.

The aim of this *ex vivo* study was to determine optimal reconstruction parameters for dental implant imaging using PCD-CT, including UHR images, VMI, and iMAR.

## Materials and methods

### Study design and ethics

Six pig mandibles were used to qualitatively and quantitatively assess the most effective PCD-CT artifact reduction methods for dental implant imaging. Each mandible received two titanium-based implants from clinically established manufacturers Dentsply, Nobel Biocare, Straumann, and Thommen Medical, randomly assigned and placed between the canine and first premolar.

Ethical approval was obtained from the Office of Animal Welfare and 3R at the University of Zurich, which confirmed that all experimental procedures complied with Swiss federal guidelines on animal research. This study adheres to the ARRIVE (Animal Research: Reporting of *In Vivo* Experiments) guidelines.

### Imaging data acquisition

The specimens were scanned using a first-generation dual-source PCD-CT system (NAEOTOM Alpha; Siemens Healthineers AG), which employs two cadmium telluride detectors. PCD-CT scans were performed at 140 kV with tin pre-filtration and a pitch factor of 0.85. The dose parameters were adjusted to match CBCT scan values [6], with a CTDI_vol_ of 3.28 mGy, a DLP of 45.9 mGy∙cm, and an effective dose of 92 μSv, using a conversion factor of 0.002 mSv∙mGy⁻¹∙cm⁻¹ [19]. Scans were reconstructed as UHR images with a slice thickness of 0.2 mm, both without and with the iMAR algorithm dedicated to dental implants. In addition, VMI were reconstructed from 70 to 190 keV in 10 keV increments at a slice thickness of 0.4 without iMAR. VMI at 130 keV were also reconstructed with iMAR following prior observations [15]. For reconstructions without iMAR, a sharp Hr76 kernel was used, while for reconstructions with iMAR, the Hr56 kernel was applied, as it represents the sharpest kernel supported by the iMAR algorithm. For all reconstructions, quantum iterative reconstruction (QIR) at level 3 was applied.

### Qualitative assessment

All image assessments were performed independently by two readers with different specializations and levels of experience: Reader A (T.T.D.), in training with 1 year of experience in oral and maxillofacial imaging; and Reader B (K.K.), in training with 4 years of experience in oral and maxillofacial imaging. Before the evaluation, a calibration session was conducted to clarify any potential ambiguities and standardize the assessment criteria. Evaluations were conducted in a blinded, randomized manner, with readers unaware of each other’s assessments, the imaging protocol, and the implant manufacturer’s specifications, to ensure an objective assessment.

A modified 5-point visual rating scale, based on the methodology proposed by Dillinger et al [20] for assessing dental implant imaging with PCD-CT, was utilized to evaluate image quality and artifact severity. Image quality was rated as follows: 5, excellent, fully diagnostic; 4, good, diagnostically sufficient; 3, moderate, limited diagnostic confidence; 2, poor, minimal diagnostic confidence; and 1, very poor, non-diagnostic.

The assessment of metal-induced artifacts associated with dental implants was performed using the following criteria [21]: 5, no or minimal artifacts; 4, minor artifacts present but without any impact on diagnostic interpretability of nearby anatomy; 3, moderate artifacts present with slight impact on diagnostic interpretability of nearby anatomy; 2, substantial artifacts present that impair diagnostic interpretability of nearby anatomy; 1, severe artifacts that heavily impair diagnostic of nearby anatomy.

### Quantitative assessment

Two experienced readers (T.T.D. and P.M.) conducted the quantitative analysis in a standardized manner.

**Fig. 1:**
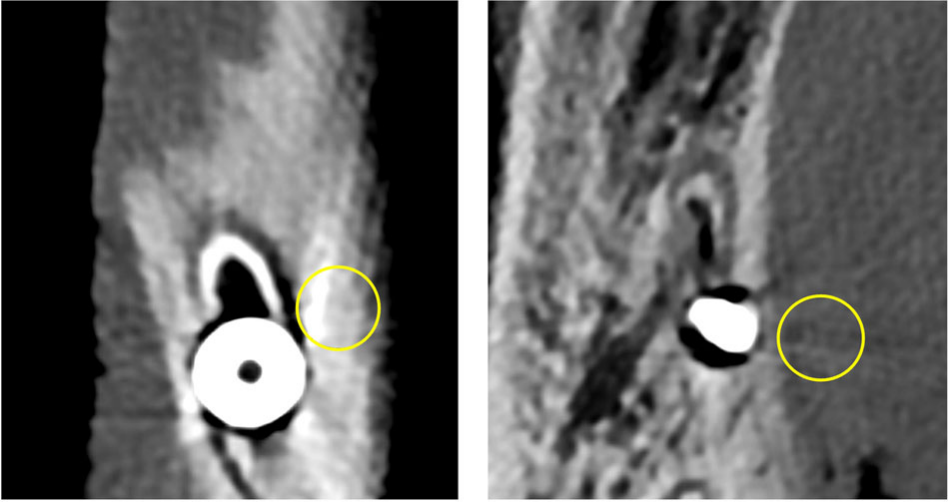
Artifact burden measurement adjacent to a dental implant. Regions of interest were placed next to the implant to capture the most pronounced hyperdense or hypodense artifacts

Artifact burden was quantified as the standard deviation (SD) of Hounsfield units (HU) within standardized peri-implant regions of interest (ROIs), as previously described [22]. ROIs were placed in anatomically comparable compartments adjacent to the implant at locations with visually apparent artifacts (Fig. 1). This metric captures the inhomogeneity of attenuation within the ROI, with higher values indicating a greater artifact burden, whereas lower values reflect more homogeneous attenuation and thus fewer artifacts. Measurements were performed for each implant on all 16 reconstructions. Artifacts are expressed as the median with interquartile range (IQR) across all implants, with lower values indicating lower artifact burden.

### Statistical analysis

Descriptive statistics were applied to summarize the data, including the calculation of medians, interquartile ranges (IQR), and the observed minimum and maximum values where appropriate. To evaluate inter-reader reliability, Krippendorff’s alpha coefficient was calculated, with values interpreted in accordance with established guidelines: a coefficient of 1.0 demonstrates perfect reliability, 0.5 indicates agreement equivalent to chance, and values below 0 suggest systematic disagreement among observers.

Quantitative analyses compared reconstruction settings (UHR images without or with iMAR; VMI from 70–190 keV in 10 keV steps without iMAR, and VMI at 130 keV with iMAR) using the SD of attenuation in peri-implant ROIs. To judge whether pooling across mandibles was appropriate, we tested between-mandible heterogeneity of SD for each reconstruction using Kruskal–Wallis tests. Friedman tests were used to assess differences between reconstructions with varying parameters. Statistical analyses were performed using IBM SPSS Statistics software (version 29.0.2.0, IBM) and in R (version 4.5.2, The R Foundation).

## Results

A total of 192 evaluations (2 implants in each of the 6 mandibles, using 16 different reconstructions) were performed.

### Qualitative results

UHR images without iMAR yielded a high image quality (median 5, for both readers; α = 1, Table 1), with minor artifact severity (median 4, for both readers; α = 1). UHR images with iMAR achieved only moderate image quality (median 3, for both readers, α = 0.758), along with minor artifact severity (median 4, for both readers) and moderate inter-reader agreement (α = 0.587).

**Table 1:** Qualitative and quantitative evaluation

Reconstructions	Image quality			Artifacts associated with dental implants			Quantitative artifact burden
	Reader A	Reader B	Inter-reader agreement (Krippendorff’s alpha)	Reader A	Reader B	Inter-reader agreement (Krippendorff’s alpha)	
UHR without iMAR	5 (5–5)	5 (5–5)	1	4 (3–4)	4 (3–4)	1	206 (156–350)
UHR with iMAR	3 (3–3)	3 (3–3)	0.758	4 (3–4)	4 (4–4)	0.587	373 (201–441)
70 keV	5 (5–5)	5 (5–5)	1	4 (3–4)	3 (3–4)	0.671	200 (146–347)
80 keV	5 (5–5)	5 (5–5)	1	4 (4–4)	3 (3–4)	0.517	171 (112–306)
90 keV	5 (5–5)	5 (5–5)	1	4 (4–4)	4 (4–5)	0.574	150 (94–274)
100 keV	5 (5–5)	5 (5–5)	1	4 (4–4)	4 (4–5)	0.574	138 (79–254)
110 keV	5 (5–5)	5 (5–5)	1	4 (4–5)	4 (4–5)	0.83	130 (69–240)
120 keV	5 (5–5)	5 (5–5)	1	5 (5–5)	5 (4–5)	0.641	125 (63–231)
130 keV	5 (4–5)	4 (4–5)	0.671	5 (5–5)	5 (5–5)	1	121 (60–224)
130 keV with iMAR	3 (3–3)	3 (3–3)	0.758	4 (4–4)	4 (4–5)	0.63	182 (61–262)
140 keV	4 (4–5)	4 (4–5)	0.83	5 (5–5)	5 (5–5)	0	119 (58–220)
150 keV	4 (4–4)	4 (4–4)	0	5 (5–5)	5 (5–5)	1	117 (57–216)
160 keV	4 (4–4)	4 (4–4)	1	5 (5–5)	5 (5–5)	1	116 (56–213)
170 keV	4 (4–4)	4 (4–4)	1	5 (5–5)	5 (5–5)	1	114 (55–211)
180 keV	4 (4–4)	4 (4–4)	1	5 (5–5)	5 (5–5)	1	114 (55–209)
190 keV	4 (4–4)	4 (4–4)	1	5 (5–5)	5 (5–5)	1	113 (55–208)

Data are median with interquartile ranges in parentheses

VMI without iMAR demonstrated a clear trend of lower artifact severity with higher VMI energy levels. Artifact severity improved from moderate to minor on VMI at 70–90 keV (median ranging from 3 to 4) to minimal on VMI at 120–190 keV (median 5, for all). Image quality was consistently excellent from 70–130 keV (median 5 from 70–120 keV; and median of 5 for reader A and median of 4 for reader B at 130 keV), except for a slight decrease to good image quality from 140–190 keV (median 4, for all). Inter-reader agreement for artifact severity varied across VMI levels (α = 0–1), with the highest concordance at 110 keV (α = 0.83) and 130 keV (α = 1). The optimal combination of high image quality and low artifact severity was observed on VMI at 120 and 130 keV.

**Fig. 2:**
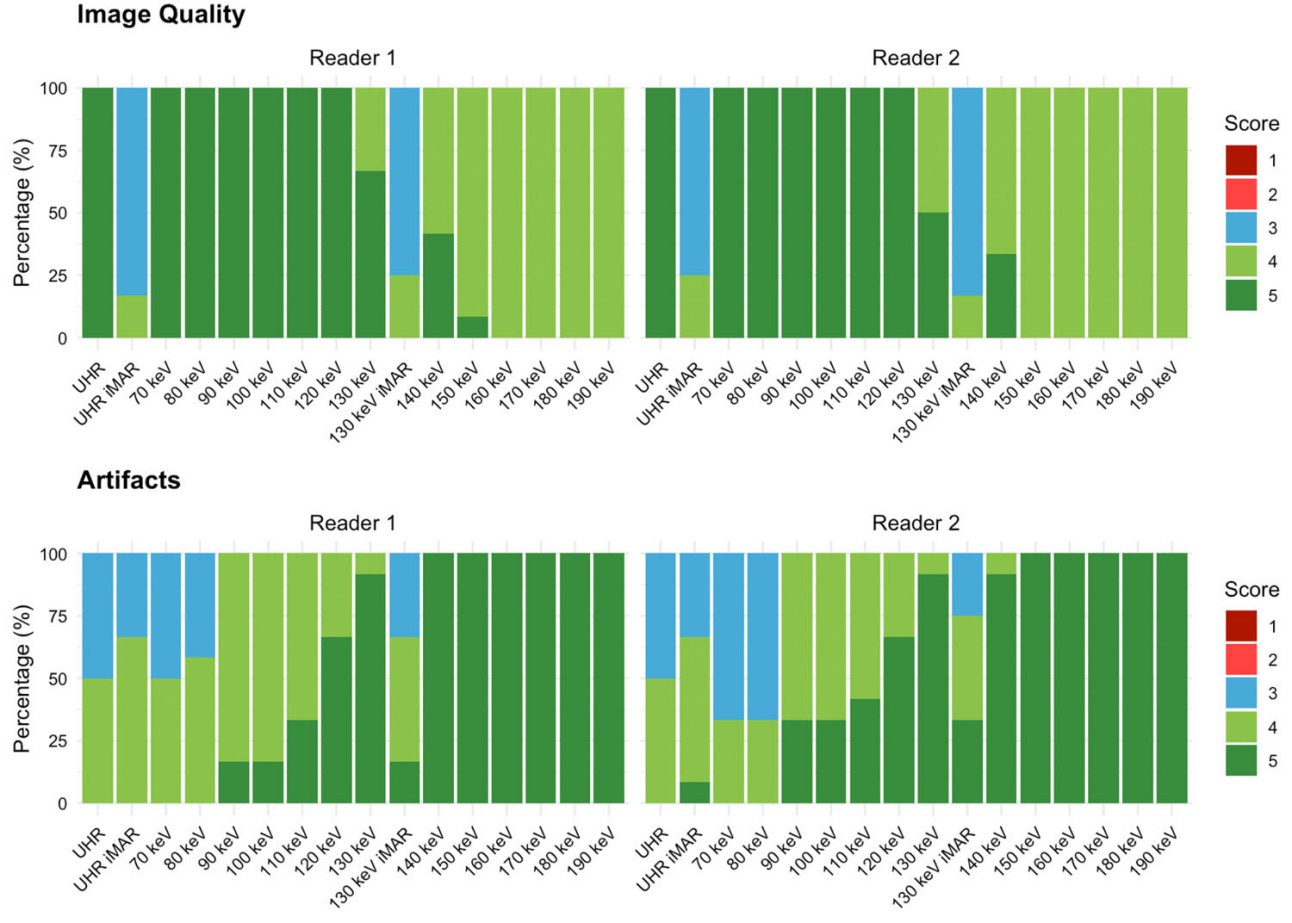
Distribution of the subjective scores assigned to each reconstruction by both readers. Ultra-high-resolution (UHR) reconstructions and virtual monoenergetic images (VMI) at 130 keV were considered the optimal combination of image quality and low artifacts for both readers. Notably, the use of iterative metal artifact reconstruction (iMAR) led to reduced image qualities for both UHR and VMI at 130 keV, while artifacts showed only minimal improvement

**Fig. 3:**
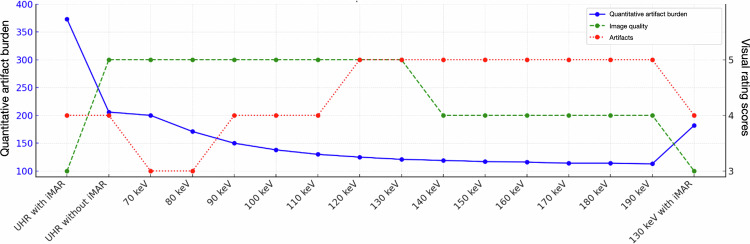
Graphical representation of the quantitative artifact burden alongside the results of the visual ratings. The optimal combination of subjective image quality, artifact reduction, and quantitative artifact burden was observed with virtual monoenergetic images at 130 keV without iterative metal artifact reduction (iMAR). The depicted values represent the median quantitative artifact burden in Hounsfield units (HU), as well as the median scores for subjective image quality and artifact ratings across both readers

**Fig. 4:**
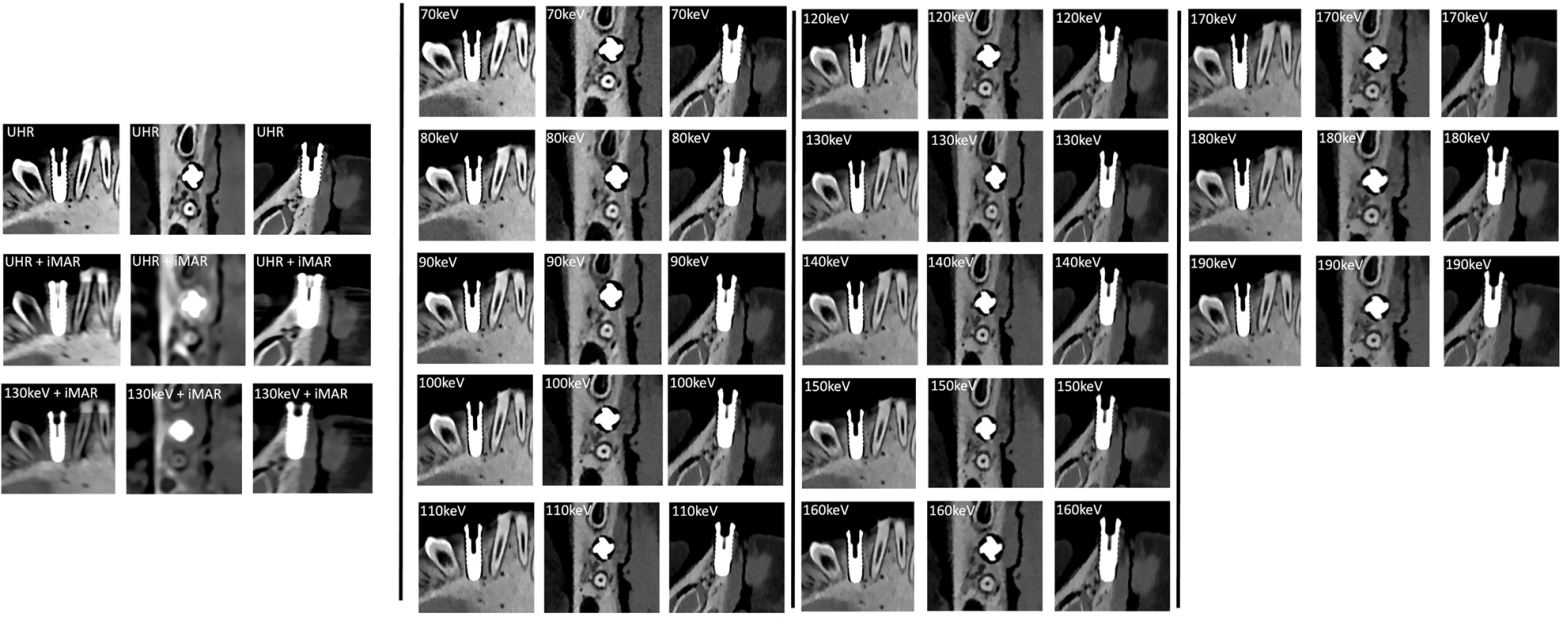
Representative visualization of an implant across various reconstructions. Scans were reconstructed as ultra-high-resolution (UHR) images and as virtual monoenergetic images (VMI) from 70 to 190 keV. UHR images provided excellent visualization of the screw threads and the bone-implant interface. In VMI, metal-related artifacts decreased at higher monoenergetic levels, with reconstructions at 130 keV deemed to offer the best image quality. The use of iterative metal artifact reduction (iMAR) with UHR images or VMI at 130 keV did not improve image quality

The combination of VMI at 130 keV with iMAR led to a lower image quality score (median 3) compared to VMI at 130 keV without iMAR and worse artifact severity (median 4, IQR 3–4 for reader A and IQR 4–5 for reader B; α = 0.63) (Figs. 2–4).

### Quantitative results

Across mandibles, artifact burden for the same reconstruction did not differ significantly (Kruskal–Wallis, *p* = 0.12–0.35), supporting pooled analyses over all 12 implants. Quantitative artifact burden decreased with increasing energy levels (Fig. 2). Thus, artifact burden decreased from 200 HU (IQR 146–347) on VMI at 70 keV to 125 HU (IQR 63–231 HU) at 120 keV and 121 HU (IQR 60–224 HU) at 130 keV, and remained similarly low up to VMI at 190 keV (113 HU, IQR 55–208 HU). At higher energies, IQR was also smaller compared to low-energy VMI, *i.e*., IQR 146–347 HU on VMI at 70 keV compared with IQR 55–208 HU on VMI at 190 keV. The combination of VMI at 130 keV with iMAR resulted in a higher artifact burden (median 182 HU, IQR 61–262 HU) compared with VMI at 130 keV without iMAR (median 121 HU, IQR 60–224 HU).

On UHR images without iMAR artifact burden was notable (median 206 HU, IQR 156–350 HU) and worsened when applying iMAR (median 373 HU, IQR 201–441 HU).

## Discussion

The objective of this *ex vivo* investigation was to determine the optimal reconstruction parameters for dental implant imaging with PCD-CT, aiming to achieve the best possible metal artifact reduction. Accordingly, 16 reconstructions were generated from each scan to evaluate UHR images, VMI at different energy levels, and the potential benefit of additional iMAR in minimizing metal-induced artifacts. Our findings indicate that UHR images and VMI at 120 to 130 keV provide the optimal balance between low artifact severity and high image quality. The additional use of iMAR did not prove beneficial for dental implant imaging.

Leveraging PCD-CT’s inherent ultra-high-resolution and spectral imaging capabilities, potentially in combination with advanced artifact reduction techniques, such as iMAR, may overcome several limitations of CBCT. These advancements improve the accurate depiction of adjacent patho-anatomical structures and significantly reduce the occurrence of artifacts caused by dental implants [23, 24].

VMI at 120–130 keV outperformed UHR images in both subjective artifact evaluation and quantitative artifact burden. We hypothesize this finding may be related to the higher image noise in UHR images at 0.2 mm compared with VMI at a slice thickness of 0.4 mm. Nevertheless, the high spatial resolution enabled an excellent delineation of the dental implants with the surrounding bone at an excellent image quality. At energy levels above 130 keV, VMI exhibited reduced tissue contrast, which limited its diagnostic value.

Considering the use of iMAR with VMI of PCD-CT, Anhaus et al demonstrated in a previous study that iMAR algorithms are effective in mitigating artifacts from dental fillings, and identified VMI at 140 keV as optimal, improving diagnostic confidence [23]. Layer et al reported significant reductions in dental implant-related artifacts in head and neck scans when using VMI from 100 to 190 keV, suggesting VMI a 130 keV as the optimal energy level [13]. In a similar study, Pallasch et al evaluated different metal artifact reduction techniques in head and neck scans in patients with dental hardware using PCD-CT. Their findings indicated superior image quality, with reduced artifacts and superior delineation of both adjacent and distant anatomical structures when combining VMI at 140 keV with iMAR compared with VMI alone [14]. Patzer et al further suggested combining VMI at 110 keV with iMAR for optimal assessment of surrounding structures in head and neck scans otherwise impaired by dental implant artifacts [21].

Although these studies recommend the combined use of VMI and iMAR for optimal artifact reduction from dental implants, their focus was primarily on surrounding soft tissues relevant to oncologic imaging rather than on applications in dentoalveolar surgery [13, 14, 21]. In the context of dedicated high-resolution dentoalveolar imaging aimed at precise assessment of the bone-implant interface, our findings indicate that the application of iMAR to UHR images or VMI does not enhance image quality or substantially reduce artifact burden. When imaging hyperdense hardware, reconstructions employing a sharp kernel enhance edge definition and result in superior image quality. However, the iMAR algorithm is currently limited to supporting only moderately sharp kernels. Therefore, we hypothesize that, in this specific imaging context, the combination of UHR images or high-energy VMI with a sharp reconstruction kernel provides superior image quality compared with images reconstructed with a moderately sharp kernel in combination with iMAR.

This study has several limitations. First, it was conducted in an *ex vivo* setting using pig mandibles, which do not fully replicate *in vivo* patho-anatomical conditions. While the findings are promising, subsequent confirmatory *in vivo* studies are mandatory. In particular, studies evaluating implant osseointegration, detecting post-implant complications such as fenestrations, dehiscence and peri-implant osteolysis, as well as assessing the clinical value of improved anatomical visualization prior to surgery in patients with existing implants are essential. Second, the variety of implant types, manufacturers, materials, and specimens was limited, restricting the generalizability of the findings across different implant geometries, materials, as well as varying dose settings that could further influence the balance between image quality and artifact suppression. Third, this study aimed at a technical comparison of reconstruction parameters rather than an evaluation of diagnostic accuracy. Future *in vivo* studies should assess the diagnostic performance in patients with the full spectrum of dentomaxillofacial implants to confirm the clinical relevance of these findings regarding metal-induced artifact reduction. Fourth, ultra-high-resolution reconstructions with a slice thickness of 0.2 mm generate large datasets, which place increased demands on storage capacity and post-processing resources. Nevertheless, in our study, these UHR images did adversely affect technical workflow efficiency.

In conclusion, this *ex vivo* study demonstrates that PCD-CT effectively reduces metal-induced artifacts in dental implant imaging, with UHR images and VMI at 120–130 keV, combined with a sharp reconstruction kernel, providing optimal image quality, whereas reconstructions with iMAR offered no further benefit.

## Data Availability

Data are available from the corresponding author upon reasonable request.

## Abbreviations

CBCT: Cone-beam computed tomography
CT: Computed tomography
HU: Hounsfield units
iMAR: Iterative metal artifact reduction
IQR: Interquartile range
PCD-CT: Photon-counting detector CT
ROI: Regions of interest
SD: Standard deviation
UHR: Ultra-high-resolution
VMI: Virtual monoenergetic images

## References

[1] Sahrmann P, Kühl S, Dagassan-Berndt D, Bornstein MM, Zitzmann NU (2024) Radiographic assessment of the peri-implant site. Periodontol 2000 95:70–86 38951952 10.1111/prd.12577

[2] Jacobs R, Salmon B, Codari M, Hassan B, Bornstein MM (2018) Cone beam computed tomography in implant dentistry: recommendations for clinical use. BMC Oral Health 18:88 29764458 10.1186/s12903-018-0523-5 PMC5952365

[3] Fleischmann D, Boas FE (2011) Computed tomography—old ideas and new technology. Eur Radiol 21:510–517 21249371 10.1007/s00330-011-2056-z

[4] Yeung AWK, Jacobs R, Bornstein MM (2019) Novel low-dose protocols using cone beam computed tomography in dental medicine: a review focusing on indications, limitations, and future possibilities. Clin Oral Invest 23:2573–2581 10.1007/s00784-019-02907-y 31025192

[5] Klintström E, Ly A, Sandborg M, Woisetschläger M, Tesselaar E (2024) Image quality of photon-counting detector CT for visualization of maxillofacial anatomy in comparison with energy-integrating detector CT and intraoperative C-arm CBCT. Eur J Radiol 181:111785 39418987 10.1016/j.ejrad.2024.111785

[6] Al-Haj Husain A, Mergen V, Valdec S et al (2025) Comparison of cone-beam computed tomography with photon-counting detector computed tomography for dental implant surgery. Int J Implant Dent 11:21 40080282 10.1186/s40729-025-00611-z PMC11906956

[7] Al-Haj Husain A, Mergen V, Valdec S et al (2025) Cone-beam *versus* photon-counting detector CT: influence of dose variations on the detection of simulated mandibular osseous lesions. J Craniomaxillofac Surg. 10.1016/j.jcms.2025.04.023 10.1016/j.jcms.2025.04.023 40393841

[8] de Boer J, Salimova N, Weidemann F et al (2025) Photon-counting CT *versus* energy-integrating detector and flat-panel CT for cadaveric wrist arthrography with additional tin filter dose reduction. Eur Radiol Exp 9:83 40883645 10.1186/s41747-025-00604-y PMC12397008

[9] Kämmerling N, Farnebo S, Sandstedt M, Booij R, Persson A, Tesselaar E (2025) Assessment of metal artifacts from titanium wrist prostheses: photon-counting *versus* energy-integrating detector CT. Eur Radiol Exp 9:45 40310571 10.1186/s41747-025-00587-w PMC12045920

[10] Mergen V, Sartoretti T, Baer-Beck M et al (2022) Ultra-high-resolution coronary CT angiography with photon-counting detector CT: feasibility and image characterization. Invest Radiol 57:780–788 35640019 10.1097/RLI.0000000000000897 PMC10184822

[11] Skornitzke S, Mergen V, Biederer J et al (2024) Metal artifact reduction in photon-counting detector CT: quantitative evaluation of artifact reduction techniques. Invest Radiol 59:442–449 37812482 10.1097/RLI.0000000000001036

[12] Hagar MT, Emrich T, Vecsey-Nagy M et al (2025) Spectral ultrahigh-resolution photon-counting CT for coronary stent imaging: evaluation in a dynamic phantom. Eur Radiol Exp 9:115 41329278 10.1186/s41747-025-00654-2 PMC12672982

[13] Layer YC, Mesropyan N, Kupczyk PA et al (2024) Use of virtual monoenergetic images for reduction of extensive dental implant associated artifacts in photon-counting detector CT. Sci Rep 14:497 38177651 10.1038/s41598-023-50926-3 PMC10766624

[14] Pallasch FB, Rau A, Reisert M et al (2024) Impact of different metal artifact reduction techniques in photon-counting computed tomography head and neck scans in patients with dental hardware. Eur Radiol 34:3742–3749 37968474 10.1007/s00330-023-10430-8 PMC11166854

[15] Sandhu S, Al-Haj Husain A, Mergen V et al (2025) Comparative evaluation of photon-counting detector CT and cone-beam CT in the assessment of simulated mandibular trauma. J Stomatol Oral Maxillofac Surg 127:102603 41067421 10.1016/j.jormas.2025.102603

[16] Al-Haj Husain A, Mergen V, Sandhu S et al (2025) Postoperative assessment of fracture reduction and osteosynthesis materials using photon-counting detector CT in maxillofacial trauma—a pilot study. Oral Maxillofac Surg 29:171 41073793 10.1007/s10006-025-01470-z PMC12513973

[17] Ruetters M, Mertens C, Gehrig H et al (2025) Dental photon-counting computed tomography for the assessment of peri-implant structures. Int J Implant Dent 11:51 40782315 10.1186/s40729-025-00640-8 PMC12335417

[18] Woisetschläger M, Booij R, Tesselaar E, Oei EHG, Schilcher J (2023) Improved visualization of the bone-implant interface and osseointegration in *ex vivo* acetabular cup implants using photon-counting detector CT. Eur Radiol Exp 7:19 37121937 10.1186/s41747-023-00335-y PMC10149426

[19] Romanyukha A, Folio L, Lamart S, Simon SL, Lee C (2016) Body size-specific effective dose conversion coefficients for CT scans. Radiat Prot Dosimetry 172:428–437 26755767 10.1093/rpd/ncv511 PMC5204364

[20] Dillinger D, Overhoff D, Froelich MF et al (2024) Photon-counting detector CT virtual monoenergetic images in cervical trauma imaging—optimization of dental metal artifacts and image quality. Diagnostics (Basel) 14:626 10.3390/diagnostics14060626 PMC10968735 38535045

[21] Patzer TS, Kunz AS, Huflage H et al (2023) Combining virtual monoenergetic imaging and iterative metal artifact reduction in first-generation photon-counting computed tomography of patients with dental implants. Eur Radiol 33:7818–7829 37284870 10.1007/s00330-023-09790-y PMC10598126

[22] Marth AA, Goller SS, Kajdi GW, Marcus RP, Sutter R (2024) Photon-counting detector CT: clinical utility of virtual monoenergetic imaging combined with tin prefiltration to reduce metal artifacts in the postoperative ankle. Invest Radiol 59:545–553 38214560 10.1097/RLI.0000000000001058

[23] Anhaus JA, Heider M, Killermann P, Hofmann C, Mahnken AH (2024) A new iterative metal artifact reduction algorithm for both energy-integrating and photon-counting CT systems. Invest Radiol 59:526–537 38193772 10.1097/RLI.0000000000001055

[24] Selles M, van Osch JAC, Maas M, Boomsma MF, Wellenberg RHH (2024) Advances in metal artifact reduction in CT images: a review of traditional and novel metal artifact reduction techniques. Eur J Radiol 170, 111276 38142571 10.1016/j.ejrad.2023.111276

